# Polymicrobial Osteomyelitis in a Patient With Isolation of *Trueperella bernardiae*: A Case Report and Literature Review

**DOI:** 10.1155/crdi/6010539

**Published:** 2025-01-28

**Authors:** Marco Antonio Delaye-Martínez, Edgar Samuel Vanegas-Rodríguez, Braulio Mendez-Sotelo, María de Lourdes García-Hernández, Claudia Adriana Colín-Castro, Rafael Franco-Cendejas, Luis Esaú López-Jácome

**Affiliations:** ^1^Department of Infectious Diseases, Instituto Nacional de Cancerología, Mexico City 14080, Mexico; ^2^PECEM, Facultad de Medicina, Universidad Nacional Autónoma de México, Mexico City 0436, Mexico; ^3^Division of Infectious Diseases, Instituto Nacional de Rehabilitación Luis Guillermo Ibarra Ibarra, Mexico City 14389, Mexico; ^4^Clinical Microbiology Laboratory, Infectious Diseases Division, Instituto Nacional de Rehabilitación Luis Guillermo Ibarra Ibarra, Mexico City 14389, Mexico; ^5^Biomedical Research Subdirection, Research Direction, Instituto Nacional de Rehabilitación Luis Guillermo Ibarra Ibarra, Mexico City 14389, Mexico; ^6^Department of Biology, Chemistry Faculty, Universidad Nacional Autónoma de México, Mexico City 04510, Mexico

**Keywords:** case report, Gram-positive rod, literature review, polymicrobial infection, *Trueperella bernardiae*

## Abstract

**Background:** *Trueperella bernardiae* is a Gram-positive rod that has been described as an opportunistic pathogen in immunocompromised patients. In a significant number of documented cases, infections with *Trueperella bernardiae* have been associated with polymicrobial infections, which highlight the fact that important bacteria–bacteria relations might be involved in the natural course of these infections, especially in patients with chronic disease courses and a history of multiple antibiotic treatments.

**Case Presentation:** We present a case of a 24-year-old woman with a 3-year history of a chronic pressure ulcer on the right foot associated with varus and cavus deformity. As per relevant medical history, she was positive for multiple wound healing sessions with wound debridement and a large number of antibiotic treatments with minimal improvement. Microbiological cultures were taken from the wound, and a soft tissue infection diagnosis was initially made. Empirical treatment was initiated with levofloxacin. At 48 h, cultures were positive for *Providencia stuartii, Pseudomonas aeruginosa, Proteus penneri, Streptococcus agalactiae*, and *Trueperella bernardiae*, and the susceptibility test was performed. Three weeks later, the symptoms progressed to purulent exudate of the wound and foul-smelling with the positive probe-to-bone test. Diagnosis was changed to polymicrobial osteomyelitis, and antibiotic therapy with ciprofloxacin and trimethoprim-sulfamethoxazole was prescribed for a 4-week course of treatment, achieving the complete remission of symptoms.

**Conclusions:** *Trueperella bernardiae* represents an emerging bacterium that can be isolated in various clinical presentations. On osteoarticular infections, the presence of comorbidities, mobility limitations, and a history of multiple antibiotic treatments may be determinant. Their isolation as part of polymicrobial infections highlights relevant interspecies interactions. Research is still lacking in determining standardized methodologies for susceptibility testing and specific clinical breakpoints to guide clinical decisions.

## 1. Background

*Trueperella bernardiae* is a Gram-positive rod, predominantly coccobacilli, non-spore-forming, non-motile, facultatively anaerobic, catalase-negative, oxidase-negative, and indole-negative bacterium, with colony diameters ranging from 0.5 mm to 1.5 mm [1–4]. It can be found as a commensal in the human skin [3–6], oropharynx [3–5], and urogenital tract microbiota [2, 3]. The pathogenic role of *T. bernardiae* in human disease has not been established due to difficulties related to its isolation [2, 6, 7], its consideration as a contaminant in cultures [2, 7], and its low frequency as a part of a pathogenic process as well [4, 8]. However, the use of matrix-assisted laser desorption/ionization time-of-flight mass spectrometry (MALDI-TOF MS) technology has allowed clinical microbiologists to identify it. Therefore, it is increasingly documented in the literature [3].

Some authors have classified this microorganism as an opportunistic pathogen [1, 9], in which the presence of comorbidities related to an immunocompromised state, such as chronic degenerative diseases [3, 10–15] or reduced mobility [7, 8, 16–18], is frequently reported. In some cases, a history of multiple surgical procedures [7, 8, 19, 20] or prolonged antibiotic treatment courses [10, 21, 22] appears to be a common factor for infection with this pathogen, especially for polymicrobial infections. Therefore, the clinical context seems to be the most relevant component of *T. bernardiae* infection.

To our knowledge, only 39 cases associated with human infection have been reported in the medical literature, which consist of 4 cases of urinary tract infection [2, 10, 19, 20], 8 cases of joint and prosthesis infection (hip and knee) [2, 7, 11, 12, 23–25], 10 cases of soft tissue and wound infection [1, 2, 4–6, 8, 13–15], 13 cases of bacteremia or sepsis [2, 3, 9, 16, 17, 21, 26–28], 2 cases of cerebral abscess [22, 29], 1 case of endocarditis [18], and 1 case of Lemièrre syndrome [30].

A notable number of articles have documented cases of *Trueperella bernardiae* infection where more than one microorganism has been involved, in which patients have long antibiotic treatments due to the presence of chronic infections. These observations suggest a potential association between *Trueperella bernardiae* and the establishment of interspecies relationships that may contribute to its pathogenicity. Nevertheless, the precise nature of these relationships remains to be established in the context of this infection.

Here, we present a case of a young woman with a history of chronic soft tissue infection that later evolved into polymicrobial osteomyelitis caused by *Trueperella bernardiae* infection. Written informed consent was obtained from the patient to publish this case report.

## 2. Case Report

A 24-year-old woman was referred to our institution because of a 3-year history of soft tissue infection in the right lower limb associated with varus foot deformity. She had received multiple antibiotic treatments and wound-healing sessions with minimal improvement. As part of her relevant medical history, the patient underwent myelomeningocele correction at birth and numerous correcting surgeries in both feet for varus and cavus deformity. The last correction surgeries were in 2002 for a tenotomy in the left lower limb and instrumentation of the right foot with placement of the osteosynthesis material in 2017. Otherwise, her past medical history was unremarkable.

Physical examination revealed varus of the right lower limb with cavus deformity of the foot. A 7 × 2 × 3 cm painful ulcer was noted in the lateral aspect of the right heel with well-defined margins, a clean bottom, and no evidence of purulent exudate. Sensitivity and range of motion were preserved without vascular involvement. The patient denied any history of fever, dizziness, nausea, or vomiting. The laboratory included a blood leukocyte and platelet count of 7700/mm^3^ and 639,000/mm^3^, respectively, C-reactive protein (CRP) levels of 2.95 mg/L, and an erythrocyte sedimentation rate (ESR) of 20 mm/hr. Due to multiple antibiotic courses, clinical history, and adverse wound evolution, it was decided to obtain a biopsy for microbiological cultures. The specimens were inoculated onto 5% blood sheep agar (BD DIFCO, Beckton, Dickinson and Company, US), MacConkey agar (BBL, Beckton, Dickinson and Company, US), both under aerobic conditions, and phenylethyl alcohol agar (PEA) (TM Media, Bhiwadi), under anaerobic conditions, at 37°C. Preliminary Gram staining of the exudate revealed abundant Gram-negative bacilli and Gram-positive cocci. A primary diagnosis of soft-tissue infection was made. The wound was initially treated locally with Prontosan (B|Braun) solution washings with surgical debridement.

**Figure 1:**
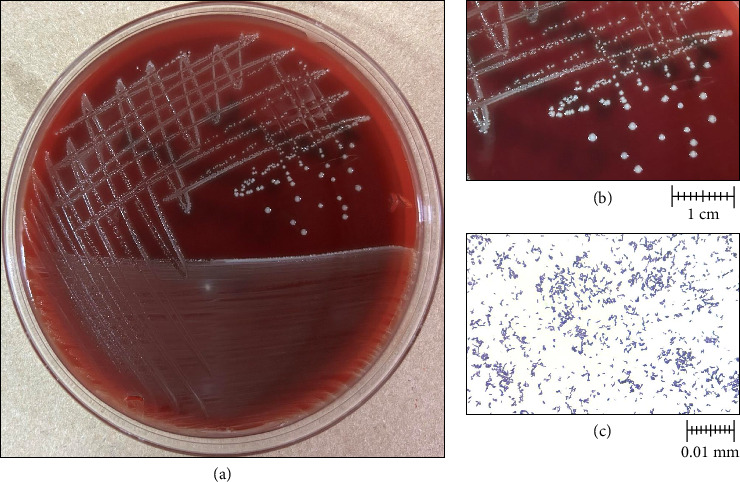
(a) Culture. *Trueperella bernardiae* culture on phenylethyl alcohol agar. (b) Amplification of *Trueperella bernardiae* colonies. Small, whitish, nonhemolytic, and rounded colonies are observed. (c) Gram staining of *Trueperella bernardiae* (100X). Gram-positive cocco-bacilli are shown.

Three Gram-negative bacilli were isolated from aerobic MacConkey agar culture, and one Gram-positive cocci was isolated from 5% blood sheep agar two days after culture incubation. Species identification with MALDI-TOF MS, Vitek MS (BioMérieux, France) technology revealed the presence of *Providencia stuartii, Pseudomonas aeruginosa, Proteus penneri,* and *Streptococcus agalactiae.* On PEA, small, whitish, nonhemolytic, and round colonies were observed (Figures 1(a) and 1(b)). Gram staining revealed Gram-positive coccobacilli (Figure 1(c)), which later were identified as *Trueperella bernardiae* by Vitek MS [31]. The identification was confirmed by Sanger sequencing targeting the 16S rRNA gene.

Minimum inhibitory concentration (MIC) for susceptibility tests was performed by the broth microdilution technique Vitek2 System (BioMérieux, MarciÈtoile, France) in all isolates, with the exception of *Trueperella bernardiae*. *Providencia stuartii* showed resistance to ampicillin-sulbactam (> 32 μg/mL) and ciprofloxacin (2 μg/mL), and *Proteus penneri* showed intermediate resistance to imipenem (2 μg/mL). *Pseudomonas aeruginosa* and *Streptococcus agalactiae* were pansusceptible.

*Trueperella bernardiae* MIC was determined by the agar dilution method in Brucella agar supplemented with hemin (5 μg/mL), vitamin K_1_ (1 μg/mL), and 5% sheep blood. As no specific clinical breakpoints have been established for resistance to *Trueperella bernardiae*, the “MIC breakpoints for Anaerobes” of the Clinical and Laboratory Standards Institute (CLSI) guides were used [32]. *Trueperella bernardiae* MIC values are shown in Table 1. Considering microbiological isolations, a severe polymicrobial soft tissue infection diagnosis was made, and empiric antibiotic treatment with levofloxacin (750 mg of PO daily for 7 days) was initiated.

Three weeks later, a medical assessment by the Infectious Diseases staff showed a purulent exudate and a foul-smelling of the wound with a positive probe-to-bone test. The antibiotic therapy was changed to ciprofloxacin (500 mg of PO q8h) and trimethoprim-sulfamethoxazole (160/800 mg of PO q8h) for 4 weeks as polymicrobial osteomyelitis was established.

Fourteen days later, the patient showed symptoms of dyspepsia and decided to discontinue treatment on a personal basis. On clinical assessment, a significant wound improvement was noted, with a reduction in size to 4 × 3 × 2 cm, cleaned margins, and no purulent exudate. The importance of continuing treatment after 1 month was emphasized to the patient. Estericide solution (ESTERIPHARMA) washings were performed with wound debridement, and KitosCell gel (Cell Therapy and Technology) and Sorbact dressings (BSN medical) were applied.

The treatment plan was accomplished, and total wound closure was observed. On physical examination, there was no evidence of exudates or fistulous tracts.

Eight months after the suspension of antibiotic treatment, the patient's progress continued to be satisfactory. The computed tomography (CT) scan had documented the absence of data suggesting infection, and the orthopedic surgeon scheduled her for right tibiotalocalcaneal arthrodesis with a retrograde intramedullary nail for cavus deformity completion of treatment.

## 3. Discussion

*Trueperella bernardiae* is described as part of the normal microbiota of the skin, oropharynx, and urinary tract [3]. Since its description in 1995, its classification has been widely changed with the emergence of next-generation sequencing technologies (NGS) [33]. Originally, the Centers for Disease Control and Prevention (CDC) classified it as “Coryneform group 2” bacteria [34], given their differences from the group 1 and group 4A-Coryneform bacteria. They tested negative for catalase, esculin hydrolysis, nitrogen reduction, triple sugar iron, and gelatin hydrolysis. Then, in 1995, *Funke* et al. [35] performed the phenotypic characterization that led to their integration into the genus *Actinomyces*. Two years later, *Ramos* et al. [33] analyzed the hypervariable regions V1-V4 of the 16S ribosomal gene and reclassified *Actinomyces bernardiae* into the *Arcanobacterium* genus. It was *Yassin* et al. [36] who in 2011 finally proposed the conformation of the *Trueperella* genus where *Arcanobacterium bernardiae* was included.

Clinical presentations of *Trueperella bernardiae* infection have been observed to vary considerably, encompassing a spectrum of manifestations ranging from severe conditions, such as necrotizing fasciitis, brain abscesses, or bacteremia, to less severe forms, including soft tissue infections, bone and prosthetic joint infections, or urinary tract infections (Table 2). Some others have reported cases of endocarditis or Lemièrre syndrome; however, these presentations are rare. On the other hand, it seems that *Trueperella bernardiae* infection occurs in an opportunistic manner, frequently associated with comorbidities, such as obesity [13, 17], type 2 *diabetes mellitus* [9, 13, 26], multiple surgical interventions [6–8, 23, 24], cancer [3, 5, 12, 15, 18], decreased functional status [27, 28], and history of past infections [21–29].

The methods used to identify *Trueperella bernardiae* have undergone significant developments in the last years. NGS has proven invaluable in differentiating between various *Trueperella* species, establishing itself as the gold standard [37]. However, the utilization of methodologies such as MALDI-TOF has proven to be beneficial, as they facilitate identification in a shorter time [31]. Following the work of Hijazin et al., the validation of databases for identifying *Trueperella bernardiae* has commenced since 2012. From this moment, almost all documented cases of *Trueperella bernardiae* infection after 2015 have employed this technology for identification with good concordance when confirming with 16S sequencing. Overall, it appears that mass spectrometry is an effective method for the identification of *Trueperella bernardiae* and should be considered for the initial approach.

*Trueperella bernardiae* natural course infection is complicated to establish, given the coexistence with polymicrobial infections. However, in patients with osteoarticular and periprosthetic infection, it appears to be associated with infection courses of more chronicity. For example, Bemer et al. [8] reported in 2009 a case of a 63-year-old man who presented for almost 30 years with recurrent episodes of infection and knee swelling. In his last relapse, both *Staphylococcus aureus* and *Trueperella bernardiae* were isolated and required surgical debridement and antibiotic therapy. Another case was reported by Otto et al. [17], involving a 78-year-old woman with a history of a pressure ulcer in the sacral region. Clinically relevant history was positive for diabetes mellitus, obesity, and multiple episodes of superinfected ulcers in both lower limbs. Three strains were isolated from the wound: *Bacteroides fragilis, Enterococcus avium*, and *Trueperella bernardiae.* The patient developed bacteremia secondary to *Bacteroides fragilis* dissemination, which later resolved with amoxicillin and clavulanic acid for 10 days. In our case, the patient had a chronic ulcer with a protracted evolution, which was previously treated on multiple occasions with antibiotic therapy with no improvement. It was also presented in the context of a polymicrobial infection. Considering the similarity to the previously mentioned cases, it is expected that *Trueperella bernardiae* infection represents a long-standing infection that might coexist with other microorganisms at the time of the diagnosis, where an immunocompromising state plays an important role in the host's susceptibility. The relationship between *Trueperella bernardiae* and anaerobic bacteria needs to be considered. In most of the cases where a polymicrobial infection was reported, other bacteria, such as *Bacteroides fragilis* [17]*, Bacteroides thetaiotaomicron* [28]*, Fusobacterium gonidiaformans* [2]*, Peptoniphilus harei* [22]*, Peptostreptococcus lacrimalis* [9], *or Actynomices* spp. [2], were also isolated. This particularity may correlate with the chronicity observed in clinical presentations that include isolates of anaerobic bacteria and the presence of *Trueperella bernardiae*, where, as noted above, it is frequently reported as part of chronic infections. Another important observation that might contribute to the polymicrobial nature of these infections is the relation between *Trueperella bernardiae* and *Staphylococcus aureus*. Almost 3 cases have been reported in the literature where both microorganisms were isolated as part of a soft tissue infection that later evolved into bacteremia. It is known that *Staphylococcus aureus* establishes relations with other microorganisms such as *Pseudomonas aeruginosa*. For example, in cases of pressure ulcers, *P. aeruginosa* is found to grow in the basal layers of the ulcer, and *S. aureus* grows more superficially [38]. By this, whether *Trueperella bernardiae* might benefit from *S. aureus* or *P. aeruginosa* warrants further investigations. Some potential mechanisms that could be involved in the development of this polymicrobial infection include the following: (i) the formation of bacterial biofilms, (ii) the presence of commensal interactions between the bacteria involved in the infection, and (iii) interspecies genetic exchange promoting antimicrobial resistance [38].

Antimicrobial treatment of *Trueperella bernardiae* infections remains a discussion topic, mainly because of the lack of consensus for antibiotic therapeutic regimens. According to literature-reported cases, the pharmacological approaches used in the susceptibility tests with the diffusion gradient epsilometry method (E-test) had been widely variable. This has resulted in difficulty to standardize MICs that are useful to establish clinical breakpoints for therapeutic decisions. Resistance to at least 16 drugs has been reported in the literature; among those that stand out are erythromycin [1, 2, 4, 7, 26], clindamycin [1, 4, 7], penicillin G [23, 26], cefotaxime [30], sulfamethoxazole-trimethoprim [4, 23, 27], amikacin [23], norfloxacin [17], daptomycin [28], phosphomycin [17], ciprofloxacin [15], levofloxacin [27], meropenem, imipenem, moxifloxacin [5], gentamicin [30], and metronidazole [11] (Supporting Table 1). This interpretation must be taken with caution because, to date, neither the EUCAST nor the CLSI guidelines have yet established specific clinical breakpoints for *Trueperella bernardiae*. Furthermore, there is no consensus regarding the most appropriate methodology for testing susceptibility. Considering this, dilution methods such as macrodilution, microdilution, or agar dilution represent a good approach for the determination of antimicrobial susceptibility for unknown clinical breakpoint bacteria, as in the case of *Trueperella bernardiae* [39]. These methods offer an advantage over epsilometry, a diffusion method, as they permit a more precise control of the inoculum size. In this sense, the number of colony-forming units (CFU) determined by McFarland densitometry and the volume to be used during the inoculation can be more accurate with dilution methods than diffusion methods, where a swab is used to spread the inoculum on the agar plate. For this reason, diffusion methods for susceptibility testing reports might be a better approach for clinical breakpoint standardization.

Despite the lack of clinical breakpoints, the therapeutic approach to *Trueperella bernardiae* infection should prioritize the selection of an antimicrobial regimen that demonstrates optimal penetration into the affected tissue and provides good coverage against other microorganisms that may accompany the infection.

All treatments used to manage infections involving *Trueperella bernardiae* have been effective. In general, the antibiotic most frequently prescribed is amoxicillin with clavulanic acid for 7–14 days, depending on the clinical context of each patient. There have been reported cases where the treatment was extended for over a month. For example, Loïez et al. [23] used a 12-week therapeutic scheme for a hip prosthetic joint infection. Pan et al. [29] used a 6-month scheme with amoxicillin to treat a 5-year-old child with a cerebral abscess, and Lawrance et al. [2] reported a 4-week scheme with amoxicillin to treat a patient with bacteremia. The treatment of *Trueperella bernardiae* infections will typically depend on the location of the infection, the presence of a monomicrobial or polymicrobial infection, and the susceptibility of the isolates. In consideration of the cases that have been published, the recommended treatment duration for bone and periprosthetic joint infections is between six and eight weeks [1, 7, 11, 23–25]. The use of quinolones [7], clindamycin [11], tetracyclines [24], or carbapenems [25] has been demonstrated to be adequate for the treatment of these infections. In contrast, the use of beta-lactams and beta-lactam inhibitors such as amoxicillin with clavulanic acid has been demonstrated to be an effective approach for the treatment of soft tissue infections [1, 2, 4–6, 8, 14, 15], urinary tract infections [2, 10, 19, 20, 27], and bacteremia [2, 3, 9, 12, 16, 21]. The recommended duration of treatment for these infections is 2–4 weeks, respectively. In the case of central nervous system infections, the use of 3^rd^ generation cephalosporins and amoxicillin has been effective with a treatment duration of 3–6 months [22, 29]. Just one case of abdominal necrotizing fasciitis has been documented, in which treatment with piperacillin-tazobactam was effective [13].

In our case, *Trueperella bernardiae* showed resistance to clindamycin (MIC ≥ 8 μg/mL) and metronidazole (MIC ≥ 32 μg/mL), according to the CLSI clinical breakpoints for anaerobes, and resistance to levofloxacin (MIC 2 μg/mL) and ciprofloxacin (MIC 4 μg/mL), given the EUCAST PK/PD for nonrelated species (Table 1). With this in mind, the patient initially received levofloxacin for 7 days. Then, because of the worsening of symptoms, the treatment was switched to ciprofloxacin and sulfamethoxazole-trimethoprim for 4 weeks until complete remission was achieved. It is well established that fluoroquinolones are recommended for the treatment of pressure ulcers or ulcers associated with venous insufficiency [40], as they provide good coverage against *Staphylococcus aureus* and gram-negative bacilli. Furthermore, in the context of bone involvement, they are an excellent option due to their ability to penetrate in such tissue [41]. The initial failure in our case was likely attributed to the polymicrobial nature of the infection. We opted for ciprofloxacin switching because of its well-known antipseudomonal activity and its effectiveness against *Proteus* species, while trimethoprim-sulfamethoxazole was selected for its efficacy against both *Streptococcus* and *Proteus* species. In addition to levofloxacin, antibiotics from the macrolide family, such as azithromycin, and first-generation cephalosporins, such as cephalothin, teicoplanin, or gentamicin, can also be useful.

Despite the resistance observed in the *Trueperella bernardiae* isolate, treatment was sufficient to achieve the complete remission of symptoms up to 8 months after treatment discontinuation.

## 4. Conclusion

*Trueperella bernardiae* is a variable hemolytic, facultatively anaerobic, Gram-positive, rod-shaped bacterium that can be found in patients with an immunocompromised state. Its isolation in the context of a polymicrobial infection, is a common finding among the medical literature, and a long course of treatment might be necessary for complete remission of the symptoms. The use of the MALDI-TOF MS system for bacterial identification appears to be effective. There is a lack of consensus on the best method to determine the susceptibility testing for *Trueperella bernardiae,* and this can be seen reflected in the lack of standardized clinical breakpoints for clinical decisions. So, it is important to define which method is better to homogenize the data reported in similar cases. Moreover, the antimicrobials to be tested in this type of assay are still needed, which might be solved if intrinsic resistance mechanisms are evaluated experimentally.

## Figures and Tables

**Table 1:** Antimicrobial susceptibility testing for the *Trueperella bernardiae* isolate.

Antibiotic	MIC (μg/mL)	Antibiotic	MIC (μg/mL)
Penicillin	S (≤ 0.25)	Ertapenem	S (≤ 2)
Ampicillin	S (≤ 0.25)	Amikacin	R (≥ 8)
Ampicillin/sulbactam	S (≤ 4/2)	Ciprofloxacin⁣^∗^	R (2)
Amoxicillin/clavulanate	S (≤ 2/1)	Levofloxacin⁣^∗^	R (4)
Ceftriaxone	S (≤ 8)	Vancomycin⁣^∗∗^	NA (≤ 2)
Cefoxitin	S (≤ 8)	Piperacillin/Tazobactam	S (≤ 16/4)
Imipenem	S (≤ 2)	Metronidazole	R (≥ 32)
Meropenem	S (≤ 2)	Tetracycline	S (≤ 2)

*Note:* The agar dilution method was used for minimum inhibitory concentration (MIC) determinations. Susceptibility interpretation was guided by “MIC breakpoints for Anaerobes” of the Clinical and Laboratory Standards Institute (CLSI).

⁣^∗^Susceptibility interpretation guided by EUCAST PK/PD (non-related species) clinical breakpoints.

⁣^∗∗^Clinical breakpoints for MIC interpretations are not standardized for this antibiotic by any guide.

**Table 2:** Clinical presentation and relevant clinical history of *Trueperella bernardiae* infections reported in the literature (1996–2024).

Author	Sex/age	Diagnosis	Clinical characteristics	Sample	Culture	Isolates	Identification method	Treatment
Ieven et al. 1996 (Belgium) [19]	69 years/male	Urinary tract infection/perirenal abscess/sepsis	P: fever, leukocytosis, bilateral hydronephrosis, nephrolithiasis, and left perirenal abscess. Not pyuria or bacteriuria	Urine from nephrostomy and blood cultures.	Both positive at 72 h	*Actinomyces bernardiae*	Biochemical identification	Amoxicillin/clavulanic acid, 13 days
			RCH: incomplete neurinoma resection in 1975, 1982, and 1985; neurogenic bladder dysfunction; and uretero-ileocutaneostomy in 1987					

Adderson et al. 1998 (US) [11]	19 years/female	Right hip infection and avascular necrosis	P: 3 days history of right hip and knee pain, fever, limitation of motion, and deteriorating renal function	Right hip aspirate	Positive at 5 days	*Arcanobacterium bernardiae*	Fatty acid profile/biochemical identification	Clindamycin, 6 weeks
			RCH: 4-year history of treatment with corticosteroids and cyclophosphamide for glomerulonephritis secondary to systemic lupus erythematosus and avascular necrosis of left hip					

Lepargneur et al. 1998 (France) [20]	75 years/male	Urinary tract infection	P: history of right back pain, fever, dilatation of inferior right ureter, calcified stones	Urine from nephrostomy	Positive at 48 h of incubation	*Arcanobacterium bernardiae*	API coryne system/biochemical identification/16S sequencing	First: Netilmicin, cefixime, 5 days
			RCH: urothelial carcinoma in1985, radical cystoprostatectomy, ureteral duplication, and ureteroileal anastomosis					Second: amoxicillin, time not specified

Bemer et al. 2009 (France) [8]	63 years/male	Infection in the lower lib	P: Persistent wound drainage in the left knee	Peroperative specimens	Positive at 96 h	*Arcanobacterium bernardiae/Staphylococcus aureus*	API coryne system/biochemical identification	Clindamycin and fusidic acid, 3 weeks
			RCH: tuberculosis arthritis of the knee, arthrodesis and femur lengthening, avascular necrosis of the bone and sequestrum, Pipeneau's technique, and bone grafting. For 30 years, recurrent swellings, which required surgical debridements and multiple antibiotic therapies					

Loïez et al. 2009 (France) [23]	78 years/male	Prosthetic joint infection (hip)	P: left lower limb hematoma secondary to trauma	Hematoma, muscle, femur, and acetabulum	Positive at 48 h	*Arcanobacterium bernardiae*	API coryne strip/16S sequencing	Rifampicin, ofloxacin, 12 weeks
			RCH: left total hip prosthesis 27 years previously. No chronic diseases					

Clarke et al. 2010 (USA) [13]	62 years/female	Abdominal necrotizing fasciitis	P: 3 days history of left lower quadrant abdominal pain, redness of the skin, tender mass on the skin, and fever	Purulent drainage	Positive at 48 h	*Arcanobacterium bernardiae/Morganella morganii*	16 S sequencing	Vancomycin, aztreonam, piperacillin-tazobactam, time not specified
			RCH: type 2 DM, neuropathy, obesity, obstructive sleep apnea, hypertension, hyperlipidemia, COPD, osteoarthritis, depression, and tinea pedis					

Sirijatuphat et al., 2010 (Thailand) [10]	60 years/male	Urinary tract infection (kidney abscess and perinephric abscess) and thoracic empyema	P: 3-week history of fever, dysuria, left loin pain, and weight loss. 1-week prior admission progressive breathlessness	Purulent drainage and pleural effusion	Not specified	*Arcanobacterium bernardiae*	16S sequencing	First: ceftriaxone, 30 days Second: clindamycin, 3 months
			RCH: poor controlled type 2 DM and several years of left renal stones					

Weitzel et al. 2011 (Chile) [16]	72 years/female	Bacteremia secondary to sacral pressure ulcer infection	P: 2 days history of fever, chills, anorexia, and progressive prostration. At admission: hypotension, tachycardia, tachypnea, mental impairment, and sacral pressure ulcer	Blood specimens from surgical debridement	Both positive at 48 h	Blood: *Arcanobacterium bernardiae* Wound: *Staphylococcus aureus* methicillin- resistant and *Citrobacter freundii*	API coryne system/16S sequencing	Amoxicillin/clavulanic acid, 6 days
			RCH: Alzheimer disease, hospitalization secondary to chronic pressure ulcers, and infection 6 months prior presentation					

Otto et al. 2013 (France) [17]	78 years/female	Sacral pressure ulcer and bacteremia	P: fever (40°C), tachycardia, polypnea, and sacral ulcer	Sacral ulcer, urine, and blood	Ulcer: 48 h Urine: 48 h Blood culture: 96 h	*Trueperella bernardiae/Bacteroides fragilis/Enterococcus. avium*	16S sequencing	Amoxicillin/clavulanic acid, 10 days
			RCH: obesity, superinfected chronic pressure ulcers in lower limbs, and multiple antibiotic treatments					

Parha et al. 2015 (UK) [22]	68 years/female	Brain abscess	P: confusion, vomiting, slurry speech, and double incontinence	Purulent drainage	Not specified	*Trueperella bernardiae/Peptoniphilus harei*	16S sequencing	Ceftriaxone, 12 weeks
			RCH: diabetes insipidus, rheumatoid arthritis, long-standing chronic suppurative otitis media, mastoiditis, and multiple antibiotic schemes					

Schneider et al. 2015 (Denmark) [9]	45 years/male	Bacteremia secondary to diabetic foot infection	P: diabetic foot, pressure ulcers in lower limbs, fever (38.2°C), and tachycardia	Purulent discharge	Positive at 48 h	*Trueperella bernardiae/Peptostreptococcus lacrimalis*	MALDI-TOF MS/16S sequencing	Amoxicillin, 14 days
			RCH: obesity, type 2 DM, atherosclerosis, neuropathy, chronic foot ulcers, and amputation of two toes of the right foot					

Gilarranz et al. 2016 (Spain) [7]	73 years/female	Prosthetic joint infection (knee)	P: chronic pain of the left lower limb, hematoma in the left knee, and no fever	Synovial fluid	Positive at 48 h	*Trueperella bernardiae*	MALDI biotyper/VITEK MS	Ciprofloxacin, 14 days
			RCH: bilateral knee osteoarthritis, bilateral total knee replacement, surgical site infection, and patellar tendon necrosis					

Rattes et al. 2016 (Brazil) [1]	24 years/female	Surgical site infection in laparoscopic cholecystectomy	P: fever, abdominal pain, and periumbilical purulent discharge	Purulent discharge	Positive at 96 h	*Trueperella bernardiae*	MALDI-TOF	Amoxicillin/clavulanic acid, 7 days
			RCH: laparoscopic cholecystectomy 7 days before					

VanGorder et al. 2016 (USA) [14]	77 years/female	Soft tissue infection (abscess)	P: 2 weeks indurated lesion on the back, painful, with purulent secretion	Purulent discharge	Positive at 48 h	*Trueperella bernardiae*	MALDI-TOF MS	Sulfamethoxazole-trimethoprim, 10 days
			RCH: lesion 2 years before with the same characteristics; resolved with antibiotic empiric treatment. No chronic diseases					

Cobo et al. 2017 (Spain) [15]	69 years/female	Surgical site infection	P: fever, pain, and purulent discharge on the surgical site	Purulent drainage	Positive at 72 h	*Trueperella bernardiae*	MALDI-TOF MS/16S sequencing	Amoxicillin/clavulanic acid, 7 days
			RCH: colostomy relocation 7 days before the presentation, rectal cancer, multiple abdominal surgeries, and recurrent episodes of pericolostomy eventration					
	70 years/female	Soft tissue infection (inguinal granuloma)	P: fever, pain, and purulent discharge	Purulent discharge	Positive at 24 h	*Trueperella bernardiae/Escherichia coli*	MALDI-TOF/16S sequencing	Amoxicillin/clavulanic acid, 7 days
			RCH: metastatic ovarian cancer, chemotherapy schemes (carbo-taxol, caclyx + carbo-taxol + topotecan), and resection of inguinal adenopathies					

Gowe et al. 2018 (US) [24]	57 years/male	Olecranon bursitis	P: fever and purulent discharge of right olecranon	Intraoperative specimens	Positive at 24 h	*Trueperella bernardiae*	Vitek 2/MALDI-TOF	Doxycycline, 14 days
			RCH: hypertension, gout, bursectomy, and tenotomy 2 years before presentation, farmer					

Lawrence et al. 2018 (UK) [2]	45 years/male	Bacteremia secondary to septic thrombophlebitis in a IV drug user	P: fever, tachycardia, and necrotic abscess at the injection site. Multiple purulent lesions in the thigh and calves	Purulent discharge and thrombus	Positive at 48 h	*Trueperella bernardiae*	MALDI.TOF MS/16S sequencing	Amoxicillin plus amoxicillin/clavulanic acid, 4 weeks
			RCH: IV drug user (heroin and cocaine), lick needle prior IV injection					
	56 years/male	Bone infection	P: Not specified	Bone specimen	Not specified	*Trueperella bernardiae*	MALDI-TOF MS	Not specified
			RHC: Diabetic foot infection					
	42 years/female	Urinary tract infection	P: Not specified	Urine	Not specified.	*Trueperella bernardiae/Enterococcus faecalis*	MALDI-TOF MS	Not specified
			RCH: Ileal conduit					
	32 years/female	Soft tissue infection	P: Not specified	Purulent discharge	Not specified	*Trueperella bernardiae*	MALDI-TOF MS	Not specified
			RCH: Breast abscess					
	42 years/male	Bone infection	P: Not specified	Bone specimen	Not specified.	*Trueperella bernardiae/Citrobacter koseri/Corynebacterium* sp.	MALDI-TOF MS	Not specified
			RCH: Diabetic foot infection					
	43 years/male	Bacteremia	P: Not specified	Blood culture	Not specified	*Trueperella bernardiae/Fusobacterium gonidiaformans/Actinomyces funkei*	MALDI-TOF MS	Not specified
			RCH: Septic thrombophlebitis in the injection drug user					
	50 years/female	Bacteremia	P: Not specified	Blood culture	Not specified.	*Trueperella bernardiae/Escherichia coli*	MALDI-TOF MS	Not specified
			RCH: Metastatic cervical cancer					
	32 years/male	Bacteremia	P: Not specified	Blood culture.	Not specified.	*Trueperella bernardiae/Fusobacterium gonidiaformans*	MALDI-TOF MS	Not specified
			RCH: Still disease					

Najwa et al. 2018 (USA) [18]	70 years/female	Polymicrobial endocarditis	P: 2 weeks history of low-grade fever and fatigue. At ER, hypotension and supraventricular. Catheter insertion erythematosus	Blood culture (central and peripheral)	Not mentioned	*Trueperella bernardiae/Globicatella sanguinis*	MALDI-TOF MS/sequencing	Meropenem and gentamicin, 6 weeks
			RCH: cervical cancer, stent failure, and end-stage renal disease. Stents changed 3 months prior to presentation					

Calatrava et al. 2019 (Spain) [4]	39 years/female	Soft tissue infection (abscess)	P: 10 days history of right breast pain and swelling	Purulent discharge	Positive at 48 h	*Trueperella bernardiae/Actinotignum sanguinis*	MALDI-TOF MS	Amoxicillin/clavulanic acid, 10 days
			RCH: No chronic diseases					

Pan et al. 2019 (US) [29]	5 years/male	Cerebral abscess secondary to suppurative otitis media	P: On the fifth day after bilateral tympanostomy: left otalgia, emesis, fever, and tonic-clonic movements	Purulent discharge form tympanostomy and cerebral abscess	Positive at 7 days	Abscess: *Trueperella bernardiae/Actinomyces europaeus* Left ear: *Trueperella bernardiae* *Corynebacterium amycolatum/Corynebacterium aurimucosum*	MALDI-TOF MS	First: meropenem, 6 weeks. Second: vancomycin, cefepime, metronidazole, 6 weeks Third: amoxicillin, 6 months
			RCH: acute-on-chronic left otitis media, bilateral tympanostomy tube placement, and multiple courses of antibiotics					

Roh et al. 2019 (Korea) [26]	83 years/female	Bacteremia	P: fever, hypotension. *K. pneumoniae* UTI 3 days prior presentation	Blood culture	Positive at 96 h	*Trueperella bernardiae/Staphylococcus aureus*	MALDI-TOF MS/16 sequencing	Teicoplanin, 11 days
			RCH: type 2 DM, cerebrovascular disease, and paroxysmal atrial fibrillation					

Casanova, et al. 2019 (Spain) [5]	43 years/female	Soft tissue infection (abscess)	P: 2 days history of fever, pain, and swelling of the right lower limb	Purulent discharge	Positive at 48 h	*Trueperella bernardiae*	MALDI-TOF MS	Ciprofloxacin, 14 days
			RCH: 7 years history of chronic myeloid leukemia treated with nilotinib					

Tang et al. 2021 (US) [6]	71 years/male	Soft tissue infection (abscess)	P: Pain, rubor, and swelling and purulent discharge from surgical scar on his right hip. A bulge was noticed 6 weeks before the presentation	Purulent drainage, and swabbing of fat and soft tissue form surgery	Not specified	*Trueperella bernardiae*	MALDI-TOF MS	First: doxycycline, 7 days + vancomycin calcium sulfate beads Second: ceftriaxone, 6 weeks Third: cefadroxil, 1–2 years
			RCH: Right hip arthroplasty performed 2 years before presentation					

Casale et al. 2021 (Italy) [3]	78 years/female	Bacteremia secondary to gynecological surgery	P: fever and 5 days history of abdominal pain. Total vulvectomy with bilateral inguinofemoral lymphadenectomy was performed 2 months prior presentation	Swabbing from surgical wound	Positive at 26–74 h	*Trueperella bernardiae/Bacteroides fragilis/Enterococcus avium*	MALDI-TOF MS/16S sequencing.	Clindamycin plus metronidazole, 14 days
			RCH: breast cancer treated with chemotherapy (QUART) 6 years before presentation, keratinizing squamous carcinoma, and hysterectomy					

Stone et al. 2021 (Australia) [25]	70 years/female	Recurrent periprosthetic joint infection	P: periprosthetic joint infection 4 months after hip replacement. Symptoms not mentioned	Collection drainage	Not specified	*Trueperella bernardiae* One month later: *Escherichia coli*	Not specified	Benzylpenicillin, 6 weeks Carbapenem, 6 weeks
			RCH: not specified					

Matsuhisa et al. 2023 (Japan) [27]	94 years/female	Sepsis following acute pyelonephritis	P: high-level fever (40°C), dyspnea, chills, and hypotension. Labs: leukocytosis, high PCR levels (12.31 mg/dL), leukocyturia, and bacteriuria. CT scan showed stones in both kidneys	A: Urine culture B: Blood culture	Not specified.	A: *Pseudomonas* spp.*/Streptococcus* spp.*/Trueperella bernardiae* B: *Trueperella bernardiae*	MALDI-TOF MS/16S sequencing	First: ampicillin/sulbactam, 9 days. Cefepime, 7 days Second: cephalexin, 6 days
			RCH: hypertension, chronic heart failure, dementia, chronic kidney disease, osteoporosis, spinal cord compression fracture, and left femoral transverse fracture. Bedridden since then					

Kumai et al. 2023 (Japan) [30]	60 years/female	Otogenic variant of Lemierre syndrome	P: 6 months otorrhea and headaches and fever (40.5°C) 4 days prior presentation. At ER, hypotension, seizures, neck stiffness, and jolt accentuation. Labs showed leukocytosis, elevated PCR, and severe thrombocytopenia. Cerebrospinal fluid with neutrophilia and low glucose. MRI showed subdural empyema and sigmoid sinus thrombosis	Blood culture	At day 11	*Trueperella bernardiae*	Not specified	First: meropenem, vancomycin, 30 days Second: levofloxacin, 41 days
			RCH: no comorbidities					

Mazin et al. 2023 (USA) [28]	50 years/male	Multifactorial bacteremia in a paraplegic patient	P: several day history of abdominal pain and malaise. At ER hypotension, leukocytosis, AKI, and hyponatremia. CT scan showed nonobstructing bilateral kidney stones, right hydronephrosis, and abdominal collection	A: Blood culture B: Abdominal fluid collection	Positive at 72 h	A: *Bacteroides thetaiotaomicron/Gemella* spp.*/Escherichia coli/Trueperella bernardiae* B: *Enterococcus faecalis*	Mass spectrometry	First: vancomycin plus cefepime, 4 and 6 days Second: metronidazole, ceftriaxone, 9 and 7 days Third: ampicillin/sulbactam, 8 weeks Last: amoxicillin/clavulanic acid, 8 weeks
			RCH: paraplegia, ischial pressure ulcer, bilateral staghorn struvite calculi, nephrostomy (multiple cystoscopies and bilateral stent placement), and type 2 DM					

Chapman et al. 2023 (USA) [21]	45 years/male	Bacteremia due to PICC- associated infection in a paraplegic patient	P: severe muscle spasms, abdominal pain, nausea, and vomiting. CT scan showed left hip fluid collection	Blood culture	Positive at 72 h	*Trueperella bernardiae/Staphylococcus aureus*	MALDI-TOF	First: piperacillin/tazobactam and vancomycin, 5 days Second: piperacillin/tazobactam and vancomycin, 2 weeks and 4 weeks
			RCH: T4-T5 spinal cord injury and multiple hospitalizations due to infections that required extended courses of antibiotics					

Saleem et al. 2023 (USA) [12]	76 years/male	Pelvic osteomyelitis and sepsis	P: 3 weeks history of bilateral lower extremity weakness, lethargy, and chills. At ER, fever and hypotension	Blood culture	Positive at 48 h	*Trueperella bernardiae*	Not specified	First: piperacillin/tazobactam and vancomycin Then: vancomycin, 6 weeks
			RCH: prostate cancer, prostatectomy, chronic urinary retention with incontinence, and chronic foley with intermittent self-catheterization					

Delaye et al. 2024 (Mexico)	24 years/female	Bone infection	P: pain, 3-year history of pressure ulcer on the right heel treated with multiple antibiotic schemes. Ulcer with well-defined margins, clean bottom, and initially no presence of purulent exudate. No history of fever, sensitivity, vascular, or motion compromised	Biopsy from ulcer	Positive at 48 h	*Trueperella bernardiae/Providencia stuartii/Pseudomonas aeruginosa/Proteus penneri/Streptococcus agalactiae*	MALDI-TOF MS/16s sequencing	Ciprofloxacin, trimethoprim-sulfamethoxazole, 4 weeks
			RCH: myelomeningocele, multiple correcting surgeries for cavus foot deformity in both feet, tenotomy in the left lower limb, and instrumentation on the right foot					

Abbreviations: AKI, acute kidney injury; DM, diabetes mellitus; ER, emergency room; P, presentation; RCH, relevant clinical history.

## Data Availability

Data sharing is not applicable to this article as no new data were created or analyzed in this study.

## Supporting Information

Supporting Table 1 (part I and II) describes the susceptibility profiles of the clinical strains of *Trueperella bernardiae* reported in similar case reports consulted in the current work. The methodology employed by each author for susceptibility testing is specified at the top of the column. Additionally, the reference guidelines utilized for the interpretation of the results are at the bottom of the table.
