# Supplementary material for: Polymicrobial Osteomyelitis in a Patient With Isolation of Trueperella bernardiae: A Case Report and Literature Review
Source: Case Rep Infect Dis. 2025 Jan 28;2025:6010539. doi: 10.1155/crdi/6010539 (PMC11824385; doi:10.1155/crdi/6010539)
Supplement: Supporting Information — Additional supporting information can be found online in the Supporting Information section. [file 6010539.f1.docx]

**Supporting Table 1. *Trueperella bernardiae* susceptibility reports in the literature (part I and II)**

The table summarizes the susceptibility profiles of *Trueperella bernardiae* clinical strains from the most of the case reports consulted during this work. The susceptibility interpretations that authors employed by the authors are listed at the bottom of the table.

**Supporting Table 1. *Trueperella bernardiae* susceptibility reports in literature (part I).**

| **Antibiotic** | ***Ieven***  ***et al. ^a^***  E-test  (µg/mL) | ***Adderson***  ***et al. ^a^***  E-test  (µg/mL) | ***Loïez***  ***et al.*** ^b^  E-test  (µg/mL) | ***Clarke***  ***et al. ^a^***  E-test  (µg/mL) | ***Otto***  ***et al. ^a^***  E-test  (µg/mL) | ***Parha***  ***et al. ^a^***  Vitek2  (µg/mL) | ***Schneider***  ***et al.* ^c^**  Disk diffusion  (mm) | ***Gilarranz***  ***et al.* ^c^**  E-test  (µg/mL) | ***Rattes***  ***et al. ^d^***  Disk diffusion  (mm) | ***Cobo***  ***et al.* ^c^**  E-test  (µg/mL) |  |
| --- | --- | --- | --- | --- | --- | --- | --- | --- | --- | --- | --- |
| Penicillin G | S (0.012) | S (0.25) | **R (0.5)** | NA (0.12) | S (0.016) | S (0.016) | S (34) | S (0.023) | S (NA) | S (0.094) |  |
| Ampicillin | - | - | - | - | S (0.0064) | S (0.125) | - | S (0.032) | - | - |  |
| Ampicillin/Sulbactam | - | - | - | - | - | - | - | - | - | - |  |
| Amoxicillin | S (0.032) | - | S (≤0.016) | - | - | - | - | - | - | - |  |
| Amoxillin/Clavulanate | - | - | S (≤0.016) | - | - | - | - | S (0.047) | - | S (0.047) |  |
| Cephalotin | S (0.032) | - | S (0.016) | - | - | - | - | - | - | - |  |
| Cefazolin | - | - | - | - | - | - | - | - | - | - |  |
| Cefotaxime | - | - | S (0.032) | - | S (1.5) | S (0.032) | - | S (1.5) | - | - |  |
| Ceftriaxone | - | - | - | NA (0.12) | S (0.38) | S (0.032) | - | - | - | - |  |
| Cefuroxime | - | - | - | - | - | - | - | - | - | - |  |
| Cefoxitin | - | - | - | - | - | - | - | - | - | - |  |
| Cefepime | - | - | - | - | - | - | - | - | - | - |  |
| Imipenem | - | - | S (0.004) | - | S (0.125) | - | - | S (0.032) | - | - |  |
| Meropenem | - | - | - | - | - | S (0.030 | - | - | - | - |  |
| Ertapenem | - | - | - | - | - | - | - | - | - | - |  |
| Erythromycin | - | - | S (≤0.016) | NA (0.06) | - | - | - | **R (>256)** | **R (NA)** | - |  |
| Clarithromycin | - | - | - | - | - | - | - | **-** | **-** | - |  |
| Clindamycin | - | S (0.06) | S (≤0.016) | NA (<0.25) | - | S (0.008) | - | **R (>256)** | **R (NA)** | - |  |
| Gentamicin | - | - | **-** | - | - | S (0.25) | - | - | - | - |  |
| Amikacin | - | - | **R (16)** | - | - | - | - | - | - | - |  |
| Trimethoprim/Sulfamethoxazole | - | - | **R (16/304)** | NA (0.25/4.75) | - | - | - | - | - | - |  |
| Levofloxacin | - | - | S (≤0.016) | NA (1) | - | - | - | - | S (NA) | - |  |
| Ciprofloxacin | **R (8)** | - | - | - | S (2) | - | - | S (1) | - | **R (2.5)** |  |
| Moxifloxacin | - | - | - | - | - | - | S (31) | - | - | - |  |
| Norfloxacin | - | - | - | - | **R (NA)** | - | - | - | - | - |  |
| Vancomycin | - | S (0.25) | S (0.025) | NA (0.5) | S (0.125) | S (0.25) | - | S (0.094) | S (NA) | - |  |
| Linezolid | - | - | S (0.5) | - | S (0.5) | - | S (29) | S (1.5) | S (NA) | S (0.38) |  |
| Piperacillin/Tazobactam | - | - | - | - | - | - | S (34) | S (0.094) | - | - |  |
| Rifampicin | - | - | S (≤0.002) | - | - | - | S (34) | - | - | - |  |
| Teicoplanin | - | - | S (0.5) | - | S (≤0.016) | - | - | S (0.125) | - | - |  |
| Fosfomycin | - | - | - | - | **R (NA)** | - | - | - | - | - |  |
| Doxycycline | - | - | - | - | - | - | - | - | - | - |  |
| Cotrimoxazol | - | - | - | - | - | - | - | - | - | - |  |
| Metronidazole | - | **R (>32)** | - | - | - | - | - | - | - | - |  |
| Tetracycline | - | **-** | - | - | - | - | - | - | - | - |  |
| Daptomycin | - | **-** | - | - | - | - | - | - | - | - |  |
| ^a^Susceptibility interpretation not mentioned guideline.  ^b^Susceptibility interpretation guided by Le Comité de L’Antibiogramme de la Société Française.  ^c^Susceptibility interpretation guided by EUCAST PK/PD (non related species) clinical breakpoints.  ^d^Susceptibility interpretation guided by EUCAST breakpoints for gram positive-anaerobes.  ^e^Susceptibility interpretation guided by CLSI clinical breakpoints.z  Susceptible (S); resistant (R); non data available for MIC interpretation (NA) | | | | | | | | | | | |

**Supporting Table 1. *Trueperella bernardiae* susceptibility reports in literature (part II).**

| **Antibiotic** | ***Cobo***  ***et al.* ^c^**  E-test  (µg/mL*)* | ***Gowe***  ***et al. ^a^***  Agar dilution  (µg/mL) | ***Lawrance***  ***et al. ^a^***  E-test  (µg/mL) | ***Calatrava***  ***et al.* ^c^**  E-test  (µg/mL) | ***Roh***  ***et al.*** ^e^  E-test  (µg/mL) | ***Casanova***  ***et al.* ^c^**  E-test  (µg/mL) | ***Casale***  ***et al.* ^c^**  E-test  (µg/mL) | ***Mazin et al. ^a^***  Broth microdilution (µg/mL) | ***Matsuhisa***  ***et al.*** ^e^  Disk diffusion  (mm) | ***Kumai***  ***et al.* ^c^**  E-test  (µg/mL) | ***Delaye***  ***et al.*** ^e^  Agar dilution  (µg/mL) |
| --- | --- | --- | --- | --- | --- | --- | --- | --- | --- | --- | --- |
| Penicillin G | S (0.064) | S (≤0.06) | S (0.004) | S (0.032) | **R (4)** | S (0.064) | S (0.064) | **I (0.25)** | S (>30) | - | S (≤0.25) |
| Ampicillin | - | - | - | - | - | - | - | - | - | S (0.12) | S (≤0.25) |
| Ampi/sulbactam | - | - | - | - | - | - | - | - | - | - | S (≤4/2) |
| Amoxicillin | - | - | - | - | - | - | - | - | S (>30) | - | - |
| Amoxi/Clav | - | - | S (≤0.016) | - | - | S (0.094) | S (0.38) | - | S (>30) | - | S (≤2/1) |
| Cephalothin | - | - | - | - | - | - | - | - | - | - | - |
| Cefazolin | - | - | - | - | - | - | - | - | S (>30) | S (0.5) | - |
| Cefotaxime | - | - | - | - | - | - | - | - | - | **R (4)** | - |
| Ceftriaxone | - | S (≤0.5) | - | - | - | - | - | S (≤0.25) | S (>30) | - | S (≤8) |
| Cefuroxime | - | - | S (≤0.016) | - | - | - | - | S (≤0.25) | - | - | - |
| Cefoxitin | - | - | - | - | - | - | - | - | - | - | S (≤8) |
| Cefepime | - | - | - | - | - | - | - | S (0.5) | - | - | - |
| Imipenem | - | - | S (0.004) | S (0.016) | - | **R (<8)** | S (0.094) | - | - | S (0.25) | S (≤2) |
| Meropenem | - | S (≤0.25) | - | - | - | **R (<8)** | S (0.25) | S (0.25) | S (>30) | - | S (≤2) |
| Ertapenem | - | - | - | - | - | **-** | S (0.094) | - | - | - | S (≤2) |
| Erythromycin | - | - | **R (>256)** | **R (1)** | **R (3)** | - | - | S (≤0.06) | S (>30) | - | - |
| Clarithromycin | - | - | **-** | **-** | **-** | - | - | - | S (>30) | - | - |
| Clindamycin | - | - | - | **R (1)** | - | - | S (0.047) | S (≤0.06) | S (>30) | - | **R (≥8)** |
| Gentamicin | - | - | - | S (1.5) | S (0.023) | - | - | - | - | **R (4)** | - |
| Amikacin | - | - | S (0.125) | - | - | - | - | - | S (>30) | - | - |
| Trimethoprim/Sulfamethoxazole | - | - | - | **R (>32)** | - | - | - | S (≤0.25) | **R (<30)** | - | - |
| Levofloxacin | - | - | - | - | - | - | - | - | **R (<30)** | I (1) | **R (2) ^c^** |
| Ciprofloxacin | **R (2)** | - | S (0.5) | S (0.5) | - | - | - | - | - | - | **R (4) ^c^** |
| Moxifloxacin | - | - | - | - | - | **R (0.38)** | - | - | - | - | - |
| Norfloxacin | - | - | - | - | - | - | - | - | - | - | - |
| Vancomycin | - | S (≤1) | - | S (0.19) | S (0.38) | - | - | S (1) | S (>30) | - | NA (≤2) |
| Linezolid | S (0.75) | - | S (0.064) | S (0.25) | - | - | - | S (1) | - | S (0.5) | - |
| Pipe/Tazo | - | - | - | - | - | S (0.38) | S (0.5) | - | S (>30) | - | S (≤16/4) |
| Rifampicin | - | - | - | S (≤0.016) | - | - | - | - | - | - | - |
| Teicoplanin | - | - | - | - | - | - | - | - | - | - | - |
| Fosfomycin | - | - | - | - | - | - | - | - | S (>30) | - | - |
| Doxycycline | - | S (0.5) | S (0.064) | - | - | - | - | - | - | - | - |
| Cotrimoxazol | - | - | S (0.016) | - | - | - | - | - | - | - | - |
| Metronidazole | - | - | - | - | - | - | - | - | - | - | **R (≥32)** |
| Tetracycline | - | - | - | - | - | - | - | S (1) | - | - | S (≤2) |
| Daptomycin | - | - | - | - | - | - | - | **R (2)** | - | - | - |
| ^a^Susceptibility interpretation not mentioned guideline.  ^b^Susceptibility interpretation guided by Le Comité de L’Antibiogramme de la Société Française.  ^c^Susceptibility interpretation guided by EUCAST PK/PD (non related species) clinical breakpoints.  ^d^Susceptibility interpretation guided by EUCAST breakpoints for gram positive-anaerobes.  ^e^Susceptibility interpretation guided by CLSI clinical breakpoints.z  Susceptible (S); resistant (R); non data available for MIC interpretation (NA) | | | | | | | | | | | |
